# Immobilization of anode-attached microbes in a microbial fuel cell

**DOI:** 10.1186/2191-0855-2-2

**Published:** 2012-01-03

**Authors:** Rachel C Wagner, Sikandar Porter-Gill, Bruce E Logan

**Affiliations:** 1 Department of Civil and Environmental Engineering, 212 Sackett Building, The Pennsylvania State University, University Park, PA 16802, USA

**Keywords:** microbial fuel cell, microbial electrolysis cell, bioelectrochemical system, immobilization layer, anode, latex

## Abstract

Current-generating (exoelectrogenic) bacteria in bioelectrochemical systems (BESs) may not be culturable using standard *in vitro *agar-plating techniques, making isolation of new microbes a challenge. More *in vivo *like conditions are needed where bacteria can be grown and directly isolated on an electrode. While colonies can be developed from single cells on an electrode, the cells must be immobilized after being placed on the surface. Here we present a proof-of-concept immobilization approach that allows exoelectrogenic activity of cells on an electrode based on applying a layer of latex to hold bacteria on surfaces. The effectiveness of this procedure to immobilize particles was first demonstrated using fluorescent microspheres as bacterial analogs. The latex coating was then shown to not substantially affect the exoelectrogenic activity of well-developed anode biofilms in two different systems. A single layer of airbrushed coating did not reduce the voltage produced by a biofilm in a microbial fuel cell (MFC), and more easily applied dip-and-blot coating reduced voltage by only 11% in a microbial electrolysis cell (MEC). This latex immobilization procedure will enable future testing of single cells for exoelectrogenic activity on electrodes in BESs.

## Introduction

Bioelectrochemical systems (BESs) are based on electron transfer between microbes and an electrode surface. Most investigations into the mechanisms of electron transfer from a microbe to an anode have focused on two microorganisms, *Geobacter sulfurreducens *(Marsili et al. 2008; Holmes et al. 2006; Strycharz et al. 2010; Inoue et al. 2010; Nevin et al. 2009; Srikanth et al. 2008) and *Shewanella oneidensis *(Bretschger et al. 2007; Gorby et al. 2006), where it has been shown that specific genes and proteins are involved in exogenous electron transfer. Further study of current-generating (exoelectrogenic) bacteria and biofilms will benefit from isolating and identifying other microorganisms that are capable of electron transfer to an electrode.

Isolation techniques to identify novel exoelectrogens have typically involved dilution-to-extinction in BESs, or isolation on ferric iron agar plates. A U-tube reactor was developed (Zuo et al. 2008) that would allow a single microbe, obtained by serial dilutions, to deposit by sedimentation onto a flat anode surface. This technique was used to identify novel exoelectrogens *Ochrobactrum anthropi *YZ-1 (Zuo et al. 2008) and *Enterobacter cloacae *FR (Rezaei et al. 2009). However, the cumbersome process required many serial transfers to obtain these isolates. A microbe related to *Clostridium butyricum *was isolated from a microbial fuel cell (MFC) using ferric iron agar plates (Park et al. 2001), but this method of isolation does not target all exoelectrogens as some microbes have been isolated that can generate current but not reduce iron (Kim et al. 2004; Zuo et al. 2008).

In addition to spread-plating techniques, screening of arrays of microorganisms on ferric iron agar plates is possible through printer technology (Ringeisen et al. 2009). This approach can be used to print very small droplets of a cell suspension diluted to contain single microbes. To take advantage of this technology, for example by printing single cells in a grid pattern onto an electrode for isolation, a robust immobilization layer is required to bind the cells to the electrode so that they do not move after application to the electrode surface. This layer should not interfere with the ability of microbes to transfer electrons to an electrode surface, or with the diffusion of substrate to the cells. Latex films were evaluated here to see if they could be used to fulfill these requirements. Latex films have previously been used to entrap microbes on non-conducting surfaces, producing a high density of organisms in a thin film that survived freezing and drying (Gosse et al. 2007; Lyngberg et al. 1999; Flickinger et al. 2007). We show here effective entrapment of bacteria-sized particles using fluorescent microspheres, and demonstrate that latex entrapped anode biofilms allow exoelectrogenic activity.

## Materials and methods

Latex was applied to two different types of anodes, carbon paper (without wet proofing; E-Tek) or graphite blocks (Grade GM-10; GraphiteStore.com Inc.), in two different types of BESs in order to evaluate the immobilization method under different conditions. Carbon paper was used as the anode in a single-chamber 28-mL microbial fuel cell (MFC) reactor with a platinum-catalyzed air cathode (Cheng et al. 2006; Liu and Logan 2004) (both electrodes with projected surface area of 7 cm^2^). Graphite blocks (projected surface area of 4.6 cm^2^) were used as anodes for a single-chamber 5-mL microbial electrolysis cells (MECs) with a 1.0 × 1.5 cm^2 ^304 stainless steel 90 × 90 mesh cathode (Call and Logan 2011). Carbon paper (projected surface area of 3.0 cm^2^) was also used as anode material in some 5-mL MECs. All reactors were inoculated using cell suspensions from pre-acclimated MFCs that were originally inoculated with domestic wastewater and acetate. A multimeter (2700, Keithley Instruments, Inc.) was used to monitor the voltage across an external resistor (*R_ex _*= 10 Ω, MEC; 1000 Ω, MFC). A power source (3645A, Circuit Specialists, Inc.) was connected to the MEC circuit to add -0.7 V to the cathode. All BESs were maintained at 30°C.

MFC medium was 100 mM phosphate buffer with 17 mM acetate as the substrate (per L: 0.62 g NH_4_Cl, 4.9 g NaH_2_PO_4_·H_2_O, 9.15 g Na_2_HPO_4_, 0.26 g KCl, 1.4 g sodium acetate, and Wolfe's vitamins and minerals) (Lovley and Phillips 1988). MEC medium was 30-mM bicarbonate buffer with 10-mM acetate as the substrate, based on the ATCC recipe for *G. sulfurreducens*, #1957 (per L: 1.5 g NH_4_Cl, 0.6 g NaH_2_PO_4_, 0.1 g KCl, 2.5 g NaHCO_3_, 0.82 g sodium acetate, and Wolfe's vitamins and minerals), without the addition of the electron acceptor. MFC and MEC reactors were operated in fed-batch mode until they successively produced at least 3 equivalent batch cycles, indicating a well-established anodic biofilm.

A monodisperse latex emulsion (SF-091; Rohm & Haas) was amended with 5% glycerol to optimize the degree of coalescence and subsequent diffusivity of the film to the substrate (Lyngberg et al. 2001; Gosse et al. 2007). This solution was applied in two different ways to well-established biofilms in the different BESs by removing the anodes temporarily from the reactors. Glycerol-amended latex (referred to simply as "latex") was applied to the carbon paper biofilm from the MFC using an air brush (Paache, BearAir, S. Easton, MA; 4.5 L/min of airflow). One, three, or five layers were applied, allowing 15 minutes between each layer, and one hour after the final layer, for drying at room temperature. For the graphite blocks and carbon paper anodes from the MEC a simpler application procedure was used, where the latex was applied by dipping the blocks or paper into the latex, and excess solution was drawn off the anode with a laboratory wipe. In other experiments, the glycerol-amended latex was diluted in water to 30% to see if performance improved with a thinner layer of latex.

The effectiveness of the latex to immobilize bacteria on the anode materials was examined using several different techniques. Direct observation of individual bacteria on an electrode, when bacteria were stained using acridine orange, was not possible due to high levels of background fluorescence. Therefore, application of individual microbes on an electrode was simulated by applying droplets of fluorescent microspheres (Fluoresbrite spheres, 4.1-μm diameter, Invitrogen) to graphite electrodes. Latex was applied by the dipping method described above. After drying, the latex-coated electrode was immersed in MFC media to simulate the electrode in a BES. The droplets were observed with fluorescence microscopy before and after latex application and MFC simulation.

For SEM visualization, small sections of carbon paper anodes with exoelectrogenic biofilms with and without latex coating were mounted in cryo-matrix and frozen. Thin slices were removed from the cross-section with a microtome until a smooth surface was obtained. The surface was etched with the cryo-SEM electron beam to remove ice crystals before viewing.

## Results

### Latex preparation

Application of glycerol-amended latex with the airbrush resulted in ~2.1 mg dry weight of latex per cm^2 ^anode area per layer. Application by dipping and blotting of the glycerol-amended latex onto graphite block resulted in ~5.3 mg/cm^2^/layer for 100% latex-glycerol, and 0.67 mg/cm^2^/layer for 30% latex-glycerol. On carbon paper, ~8.1 mg/cm^2^/layer was applied for 100% latex-glycerol, and 2.5 mg/cm^2^/layer for 30% latex-glycerol.

### Immobilization of microspheres and microbes

Fluorescent microspheres are often used as analogs for microorganisms (Smith and McKay 2005; Solomon and Matthews 2005). The location and shape of a droplet of microspheres (4.1-μm diameter) on an electrode were retained after latex application and drying, and after submersion in standard MFC media.

**Figure 1:**
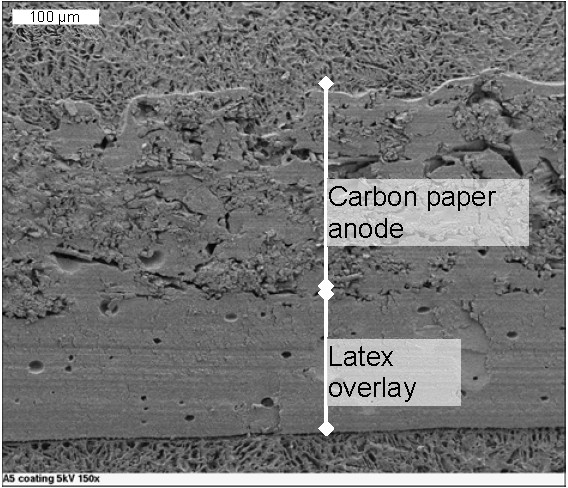
**SEM image of 3 layers of latex ("latex overlay"; approximately 165 μm thick) on a carbon paper anode with exoelectrogenic biofilm after 6 cycles in an MFC**. The biofilm is not visible due to SEM preparation techniques necessary to maintain the latex layer.

The latex film applied with an airbrush to an exoelectrogenic biofilm on a carbon paper anode remained completely intact, without dissolving or cracking, after 6 cycles in an MFC (Figure 1). The layers of latex coalesced into one continuous overlay. The biofilm was not visible in SEM images due to preparation requirements for the latex; however, the presence of the biofilm was confirmed by the exoelectrogenic activity through current production in the MFC. The latex layer applied to the MEC carbon paper anode using the dip-and-blot method also remained visibly intact throughout the experiment. The latex layer applied to the graphite block with the dip-and-blot method had variable performance. The layer made using the 30% dilution remained intact. However, at full strength, the latex layer did not consistently remain adhered to the block, and in some reactors, the latex began to peel off after ten days.

### Latex coatings on anode biofilms

**Figure 2:**
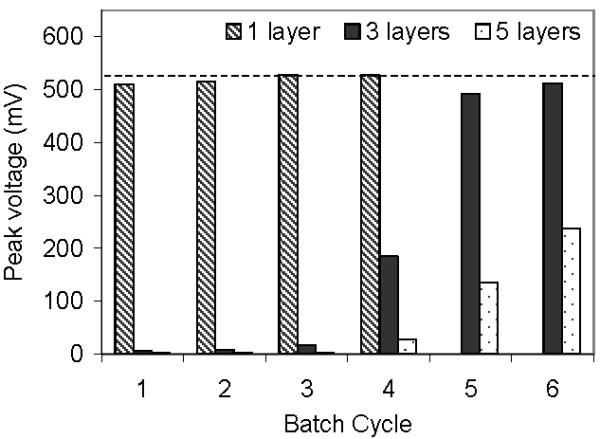
**An exoelectrogenic biofilm on a carbon paper anode in an MFC with 1, 3, or 5 layers of latex applied to a carbon paper anode using an airbrush, compared to a reactor with no latex (dashed line)**. Representative reactors are shown.

When one layer of glycerol-amended latex was applied with the airbrush to a biofilm on carbon paper in an MFC, the reactor recovered immediately to its pre-latex voltage. When three layers were applied, the reactor returned to its original performance in 6 cycles. However, when five layers were applied, the MFC only reached 45% of its original voltage even after 6 cycles (Figure 2).

**Figure 3:**
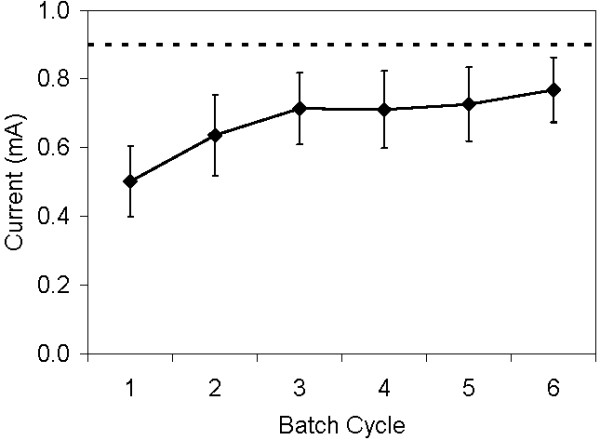
**An exoelectrogenic biofilm on a graphite block anode in an MEC immobilized with glycerol-amended latex diluted to 30% strength, compared to the biofilm with no overlay (dashed line)**.

Using undiluted glycerol-amended latex for immobilization of microbes on a graphite block, the MEC with graphite block anode returned to 42% (± 8%) of its original current within three cycles of latex application by dipping and blotting. However, after three cycles, which took approximately 10 days, the overlay had started to delaminate from the graphite block, so testing was discontinued. Using a 30% dilution of the latex-glycerol, the current recovery in the MECs improved, reaching 85% (± 9%) of the original current within 3 cycles of latex application with consistent results over 3 additional cycles (Figure 3). In addition, the latex remained adhered to the anode.

**Figure 4:**
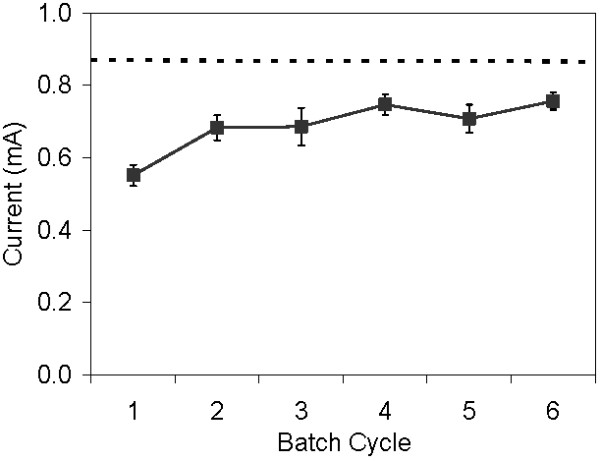
**An exoelectrogenic biofilm on a carbon paper anode in an MEC immobilized with glycerol-amended latex diluted to 30% strength, compared to the biofilm with no overlay (dashed line)**.

When the undiluted overlay was applied to carbon paper anodes in the MECs, current returned to 43% of the original level within 3 cycles and was maintained in further batches. With the thinner, 30% diluted layer, current returned to 89% (± 6%) of the original current within 3 cycles of application and remained consistent in subsequent batches (Figure 4).

## Discussion

Latex films were shown be effective in holding individual particles (fluorescent microspheres) or active biofilms on electrically conductive surfaces. Microbes trapped on two different surfaces (carbon paper and graphite block) using different application methods (airbrushing and dip-and-blot) retained most of their exoelectrogenic capability. On both surfaces, and in both MFC and MEC reactors, increasing the amount of latex applied onto the biofilm adversely affected the ability of the anode to recover exoelectrogenic activity to pre-application current levels (Lyngberg et al. (2001)). found that effective diffusivity through the latex was highly dependent on layer thickness. Therefore, this decrease in activity was likely due to a reduction in mass transfer to (substrate) and from (protons) the biofilm with thicker layers of latex.

The latex coating thickness, measured by dry weight, on the graphite block was less than that of the graphite paper, and the full strength latex coating did not stick well to the block. The coating on the carbon paper when applied by the air brush to the MFC anode or the dip-and-blot method (at 30% strength) to the MEC anode was similar (slightly more than 2 mg/cm^2^/layer). While the MFC regained 100% of its pre-application performance, the MEC was limited to about 89% of its pre-application performance. It is unlikely that there was any decrease in the performance of the MEC in these experiments due to exposure of the biofilm to oxygen during the latex application, as MEC biofilms are routinely exposed to air when it they are refilled (often intentionally to reduce methanogenesis) without adverse affects to current production (Call and Logan 2008). In addition, the biofilm in an MFC is routinely exposed to oxygen in air due to oxygen diffusion through the cathode and into the anode chamber without apparent adverse effects. If desired, the latex film could be applied under strictly anoxic conditions in an anaerobic glove box. Previous work with bio-catalytic films used for hydrogen gas production has shown that the coating itself is not adversely affected by the presence or absence of air, nor is the performance of that biofilm (Gosse et al. 2007). However, it is possible that some strict anaerobes might be affected by oxygen during this procedure, so anaerobic application of the latex biofilm may be of interest in future studies.

The ability to immobilize microbes on an electrode using a latex film has two valuable applications for BESs, but for successful application in BESs, immobilization of microbes on electrodes must not interfere with the ability of cells to transfer electrons. Bioelectrochemical features seen in cyclic voltammograms of pectin-entrapped *Geobacter *biofilms have been shown to be similar to naturally-grown *Geobacter *biofilms (Srikanth et al. 2007). This suggests that entrapment by itself is not changing the electrical capability of the cells, although they found current was somewhat decreased as observed here as well. One application of an immobilization layer for cells on a BES electrode is isolation of microbes directly on an electrode. This requires immobilization of an array of single cells, without greatly compromising current generation, which our latex overlay achieves. In addition, a biofilm of specific microbes can be developed on an electrode in a controlled setting, immobilized and protected under a latex coating, and then introduced to a more complex, non-sterile environment. Under the coating, these organisms would not have to compete with other microbes for the electron-accepting surface. Exoelectrogenic biofilm activity under a glycerol-amended latex film can be restored to nearly the same levels as pre-application activity, making it a suitable immobilization layer for these applications.

## Competing interests

The authors declare that they have no competing interests.
